# Wild-Growing and Conventionally or Organically Cultivated *Sambucus nigra* Germplasm: Fruit Phytochemical Profile, Total Phenolic Content, Antioxidant Activity, and Leaf Elements

**DOI:** 10.3390/plants12081701

**Published:** 2023-04-19

**Authors:** Theodora Papagrigoriou, Panagiota Iliadi, Milan N. Mitić, Jelena M. Mrmošanin, Katerina Papanastasi, Eleftherios Karapatzak, Eleni Maloupa, Alexia V. Gkourogianni, Anastasia V. Badeka, Nikos Krigas, Diamanto Lazari

**Affiliations:** 1 Laboratory of Pharmacognosy, School of Pharmacy, Aristotle University of Thessaloniki, 54124 Thessaloniki, Greece; 2 Laboratory of Food Chemistry, Department of Chemistry, University of Ioannina, 45110 Ioannina, Greece; 3 Department of Chemistry, Faculty of Sciences and Mathematics, University of Nis, 18000 Nis, Serbia; 4 Institute of Plant Breeding and Genetic Resources, Hellenic Agricultural Organization Demeter, 57001 Thessaloniki, Greece

**Keywords:** elderberry, DPPH, Folin–Ciocalteu, total flavonoids, cultivated germplasm, Brix, pH, vitamin C, acidity, micronutrients, macronutrients

## Abstract

European elder or elderberry (*Sambucus nigra* L., Viburnaceae) is a plant species with known high pharmaceutical and nutritional value. However, the Greek native germplasm of *S. nigra* has not been adequately utilized to date as in other regions. This study evaluates the fruit antioxidant potential (total phenolic content and radical scavenging activity) of wild-growing and cultivated germplasm of Greek *S. nigra.* In addition, nine cultivated Greek *S. nigra* genotypes were evaluated regarding the effects of fertilization (conventional and organic) on fruit phytochemical and physicochemical potential (total flavonoids, ascorbic acid content, pH, total soluble solids, and total acidity), as well as on the antioxidant potential (total phenolic content and radical scavenging activity) of fruits and leaves. Additionally, an analysis of macro- and micro-elements in the leaves of the cultivated germplasm was performed. The results demonstrated comparatively higher total phenolic contents of fruits of cultivated germplasm. The genotype was the decisive factor in the fruits’ phytochemical potential and leaves’ total phenolic content of cultivated *S. nigra* germplasm. Similarly, fertilization regime effects were found to be genotype-dependent, affecting fruit phytochemical and physicochemical attributes. The trace element analysis results were similar, with genotypes varying significantly in their concentrations of macro- and micro-elements. The current work builds on previous domestication attempts for Greek *S. nigra*, providing new data on the phytochemical potential of this important nutraceutical species.

## 1. Introduction

The genus *Sambucus* L. (Viburnaceae) includes approximately 20 species, which are naturally distributed across almost all continents (https://powo.science.kew.org/taxon/urn:lsid:ipni.org:names:30018446-2, accessed on 28 February 2023). *Sambucus nigra* L., commonly known as black elder, European elder, or elderberry, is native to the temperate regions of Europe, West Asia, and North Africa, though it has also been introduced to the Americas, South Asia, and Australia [1,2,3]. It is a perennial deciduous shrub that can reach the height of a small tree, with large, dark-green pinnate leaves and small, white–yellowish fragrant flowers arranged in corymb inflorescences that blossom through May to June [4]. The fruits of black elder are small, spherical drupes that ripen during August and September in temperate regions. Depending on the maturity stage, their colour ranges from green (for unripe fruits) to shiny black–violet (for fully ripe ones) [5]. In the wild, *S. nigra* prefers open, woody areas with ample sunlight, although it has also been cultivated during the last 40 years for commercial purposes throughout Europe and North America [2,3,6,7].

*S. nigra* is a highly revered medicinal plant that has been employed to treat a variety of ailments since antiquity. The fruits (elderberries), its flowers (elderflowers), and, to a lesser extent, the leaves of this plant have been essential materials in folk phytotherapy for centuries, and elderberry infusions are used as a diaphoretic and immunostimulatory agent against the common cold, as well as a diuretic, laxative, and anti-inflammatory agent [8]. The flowers are also prepared into infusions and gargles to treat the common cold and other respiratory tract illnesses, such as influenza and bronchitis, as well as cough, throat inflammation, and catarrh, or against abdominal pain, digestive ailments, and rheumatisms. Additionally, elderflowers are used internally as diuretic, antidiabetic, and galactagogues, and externally in topical applications for healing wounds and burns, treating conjunctivitis and other eye irritations, and mitigating rheumatic and joint pain [6,9,10]. Aside from these medicinal uses, elderberries and elderflowers are also processed into jams and jellies, syrups and concentrates, infusions, and other beverages (both alcoholic and non-alcoholic ones) due to their unique, pleasant, and complex sensory properties [7,9].

In recent decades, black elder has attracted significant research attention aimed at elucidating its phytochemical profile and investigating proof for its medicinal properties. The antiviral and antimicrobial activities of *S. nigra* fruit and flower extracts have been validated by numerous studies [8,11], resulting in two officially finalized European Medicines Agency monographs for its fruits (*Sambuci fructus*, https://www.ema.europa.eu/en/documents/herbal-report/final-assessment-report-sambucus-nigra-l-fructus_en.pdf, accessed on 26 February 2023) and flowers (*Sambuci flos*, https://www.ema.europa.eu/en/documents/herbal-report/final-assessment-report-sambucus-nigra-l-flos-revision-1_en.pdf, accessed on 26 February 2023) [12,13]. These have allowed the launch of commercially available products, while ongoing research on the potential activity of black elder against COVID-19 is currently supported by encouraging results [14,15]. Other studies also corroborate the antioxidant, anti-inflammatory, immunomodulatory, and anticancer properties of various *S. nigra* extracts [2,4,6,16,17,18,19].

The above-mentioned properties of black elder are attributed to its rich and complex phytochemical composition. which may vary depending on factors such as the cultivar, climate (light, temperature, and precipitation), soil, fertilization and cultivation methods, as well as post-harvest treatments and storage conditions [5,20,21]. Polyphenols comprise the most important category of active compounds in elderberries, with anthocyanins (such as cyanidin-3-glucoside, which is the major anthocyanin, and cyanidin-3-sambubioside), flavonols (kaempferol and quercetin, along with their respective glycosides), and phenolic acids (both hydroxybenzoic and hydroxycinnamic derivatives, such as gallic acid, protocatechuic acid, caffeic acid, etc.) being the most notable. Tannins and other organic acids, including malic, citric, shikimic, fumaric, malonic, valeric, and tartaric acids, have also been reported [2,6,22]. As far as their nutritional value is concerned, *S. nigra* fruits contain small amounts of protein (2.7–2.9%, while the respective percentages in flowers and leaves are 2.5% and 3.3%, respectively) and a high content of fatty acids, mainly linoleic and oleic acid [5,20,21]. Vitamins of the A- and B- complexes are also present in elderberries. Ascorbic acid has also been reported, although the quantitative findings are quite inconsistent [5,20,21]. Apart from organic nutritional factors, elderberries and elderflowers are also a rich source of valuable minerals (K, Ca, P, Na, and Mg) and microelements (Fe, Mn, Zn, and Cu). The concentration of essential elements can vary considerably, and these variations are attributed to the origin of the plant species, as well as to the influence of the local environment, including soil typology and climate conditions [23,24]. However, wild-growing *S. nigra* leaves, seeds, bark, and unripe berries also contain certain toxic secondary metabolites, such as sambunigrin, a cyanogenic glycoside that has been shown to be life-threatening when consumed in large quantities due to its conversion into hydrogen cyanide (HCN) [25].

Local wild-growing plants constitute a significant source of germplasm with high utilization potential in terms of domesticating new crops with high pharmaceutical and nutritional (also termed as nutraceutical when beneficial) value [26,27,28,29]. *S. nigra* represents a potential candidate for valuable germplasm resources, considering its recognized properties and nutraceutical potential [6]. A basic domestication framework of the local wild-growing germplasm of *S. nigra* has already been proposed in Greece, where the local *S. nigra* germplasm has previously been neglected and underutilized [30].

In the above-mentioned framework, the aims of this work focused on the chemical composition of elderberries and leaves, depending on the genotype and cultivation system. The fruit’s antioxidant potential was assessed for the first time in Greek wild-growing *S. nigra* individuals and was compared with that of cultivated germplasm, coupled with the determination of the macro- and micro-elements of leaves from cultivated germplasm. Subsequently, to establish a cultivation protocol for Greek *S. nigra*, the effects of distinct fertilization regimes (conventional and organic) were evaluated in terms of the phytochemical and physicochemical profiles of produced fruits. In addition, the antioxidant potentials of the leaves and fruits of conventionally and organically fertilized cultivated germplasm were assessed. The current work builds on previous domestication attempts for Greek *S. nigra*, providing new data on the phytochemical potential of wild-growing and cultivated germplasm of this species.

## 2. Results

### 2.1. Total Phenolic Content and Antioxidant Activity of Fruits in Wild-Growing and Cultivated Germplasm

The total phenolic content was found to be significantly higher in cultivated germplasm when compared with wild-growing germplasm originating from the same population samples or genotypes (Table 1, *p* = 0.009).

### 2.2. Trace Elements in Leaves of Cultivated Germplasm

The contents of macro- and micro-elements in the leaves of nine Greek *S. nigra* genotypes that were established in the cultivation trial are presented in Table 2 and Table 3, which were taken from acclimatised young trees without any fertilization treatment (controls). Cadmium (Cd), beryllium (Be), selenium (Se), thallium (Tl), mercury (Hg), and vanadium (V) were not detected because their contents were below the detection limit of the method. The results of the ICP OES analysis showed high variation among genotypes, with significant differences in the contents of all measured elements. Potassium (K) was the most abundant macro-element, with genotype GR-1-BBGK-19,425 showing the highest value (10,053.423 mg g^−1^), followed by GR-1-BBGK-19,596 (9500.091 mg g^−1^) (Table 2, *p* < 0.05). The second-most-abundant macro-element was calcium (Ca), with genotypes GR-1-BBGK-19,562 (6801.939 mg g^−1^) and GR-1-BBGK-19,192 (6628.975 mg g^−1^) showing the highest contents (Table 2, *p* < 0.05). Concerning micro-elements, genotypes GR-1-BBGK-19,192 and GR-1-BBGK-19,425 showed the highest contents of the two most abundant micro-elements, aluminium (Al) and iron (Fe) (Table 3, *p* < 0.05). As far as detectable toxic elements are concerned, the average concentrations of arsenic (As) and lead (Pb) were 0.279 µg g^−1^ and 0.541 µg g^−1^, respectively (Table 3). Both results are below the permissible limits of toxic metals in herbal medicines and products determined by the WHO/FAO [31]: a maximum of 10 mg kg^−1^ is permitted for Pb, whereas, for As, there is not a universal limit, though Canada, China, and Thailand have set 5, 2 and 4 mg kg^−1^, respectively, as the maximum permissible levels.

### 2.3. Fruit Phytochemical Profile of Conventionally and Organically Cultivated Greek Germplasm

The phytochemical profiles of fruits of conventionally and organically cultivated germplasm of nine genotypes were assessed via the total flavonoids (TF), ascorbic acid content, and the physicochemical attributes of total soluble solids (TSS, °Brix), total acidity (TA), and pH. The phytochemical and physicochemical profiles of fruits were found to vary among genotypes and among the measured attributes, and were affected by fertilization treatments. However, most differences in the measured attributes were observed among genotypes within each fertilization treatment compared with the differences between fertilization treatments within each genotype (Table 4, *p* < 0.05). For organic fertilization, the genotype GR-1-BBGK-19,574 showed the highest TF and ascorbic acid contents with 221.95 g catechin 100 g^−1^ and 2.27 mg ascorbic acid 100 g^−1^, respectively (Table 4, *p* < 0.05, *p* = 0.002 for TF and *p* = 0.006 for ascorbic acid). The effects of fertilization manifested in the physicochemical attributes of total acidity (TA) and total soluble solids (TSS, °Brix) in the genotypes GR-1-BBGK-19,425 (*p* = 0.002 for TA, *p* = 0.008, for TSS), GR-1-BBGK-19,562 (*p* = 0.04 for TA), and GR-1-BBGK-19,574 (*p* = 0.002 for TA), whereas the genotype GR-1-BBGK-19,629 was the only one that showed higher total flavonoids (TF) under organic fertilization and the control compared with conventional fertilization (*p* = 0.032, Table 4, *p* < 0.05). The genotype, rather than fertilization treatment, seemed to be the decisive factor determining the physicochemical and phytochemical attributes of *S. nigra* fruits.

### 2.4. Total Phenolic Content and Antioxidant Activity of Leaves and Fruits in Conventionally and Organically Cultivated Germplasm

**Figure 1:**
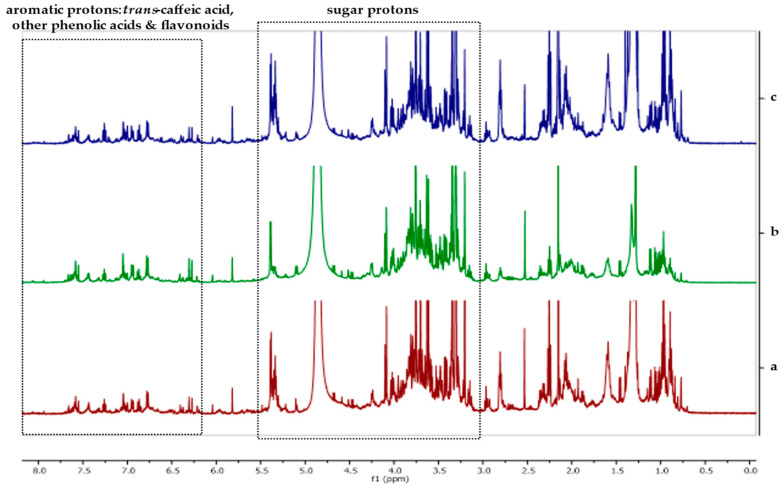
Representative ^1^H-NMR spectra of the hydromethanolic extracts of *Sambucus nigra* leaves from genotype GR-1-BBGK-19,596 under (**a**) no fertilization (control), (**b**) conventional, and (**c**) organic fertilization treatments (CD_3_OD, 500 MHz). Chemical shifts are reported in ppm on the *x*-axis (0.0–8.0).

Leaves and fruits were assessed in terms of the TPC and AA of nine genotypes herein under trial cultivation conditions, where conventional and organic fertilization regimes were applied, including a control (no fertilization). A high level of variability was observed among genotypes regarding the response of the fruit phytochemical attributes to fertilization (Table 5). Statistically significant differences in the AA and TPC of fruits were observed, mainly between genotypes under the same fertilization regime, rather than between fertilization regimes under the same genotype. Similar to TF and vitamin C, the best-performing genotype in terms of TPC under organic fertilization was GR-1-BBGK-19,574, with 375.45 mg GAE 100 g^−1^ (Table 5, *p* = 0.005). Genotype GR-1-BBGK-19,562 was the only one significantly affected by fertilization regime, with higher values of TPC under organic fertilization and the control compared with conventional fertilization (Table 5, *p* = 0.01). The antioxidant activity of fruits varied among genotypes under matching fertilization treatments, but was found to be high in most of the tested genotypes; for example, that under organic fertilization was >70% RSA in seven out of the nine tested genotypes (Table 5, *p* < 0.05). Concerning the phytochemical assessment of leaves, significant variability was observed in the TPC of leaves among genotypes under matching fertilization treatments (Table 6, *p* = 0.019); however, there were no significant fertilization treatment effects (Table 6, *p* < 0.05). On the other hand, no genotype effects were observed on the AA of leaves, which was high throughout (>90%) (Table 6). However, fertilization effects were observed for genotypes GR-1-BBGK-19,629 (Table 6, *p* = 0.038) and GR-1-BBGK-19,574 (Table 6, *p* = 0.041), with the latter performing better in organic fertilization and the control compared with conventional fertilization (Table 6, *p* < 0.05). The ^1^H-NMR spectra of leaf extracts revealed a complex phenolic profile, as all extracts were found to contain phenolic acids and flavonoids in both glycosylated and non-glycosylated forms: *trans*-caffeic acid and its derivatives were identified by the presence of a characteristic pair of *trans*-olefinic proton signals at 7.57 and 6.29 ppm (*d*, *J* = 15.9 Hz), whereas signals that corresponded to ABX systems and other aromatic protons (at 7.67–6.21 ppm) indicated the presence of other phenolic acids and flavonoids. Signals between 5.49 and 3.20 ppm were assigned to sugar protons. The study of the ^1^H-NMR spectra also revealed subtle differences between fertilization treatments within each genotype, thus corroborating the results of the in vitro analyses. In Figure 1, the ^1^H-NMR spectra of hydromethanolic extracts of *S. nigra* leaves from genotype GR-1-BBGK-19,596 under the three fertilization treatments are shown. For a further detailed presentation of the ^1^H-NMR spectra of each studied genotype, please see Supplementary Material Figures S1–S8.

## 3. Discussion

### 3.1. Fruit Antioxidant Potential of Wild-Growing and Cultivated Greek Germplasm

The antioxidant activity (AA) of fruits from wild-growing and cultivated germplasm originating from the same population samples (genotypes) of *S. nigra* was found to be high in both cases, with no statistically significant disparity, whereas cultivated Greek elderberries had a significantly higher TPC compared with their wild-growing counterparts from the same genotypes originally collected from the same location. The antioxidant activity of cultivated elder fruit extracts has been corroborated by numerous studies; however, the literature concerning the phytochemical profile of wild-growing fruits compared with cultivated ones is rather limited. Previous studies [31] have observed the fruit samples collected from the wild had lower antioxidant activity than most cultivated ones (measured using the ABTS and FRAP assays), and differences were attributed to possible lower TPCs. Other studies [32] have also reported that extracts from wild-growing *S. nigra* fruits may exhibit lower average AA and TPCs compared with cultivated samples (397.5 vs. 581.3 μmol Trolox g^−1^ DW and 5678.8 vs. 7087.3 mg ChAE 100 g^−1^ DW, respectively); the latter emphasizes the impact of environmental (edaphic and climatic) conditions on the production of phenolic secondary metabolites in black elder fruits. In addition, the maturity stage of fruits should also be considered when contrasting the phytochemical profiles of different types of *S. nigra* material [33]. Additionally, some studies [25] report that *S. nigra* plants that grow in higher altitudes, with longer exposure to intense sunlight, accumulate higher quantities of polyphenols in their individual organs. However, the cultivation conditions usually differed from those prevailing in the natural habitat of species, which, as well as the phytochemical profile, can affect plant growth patterns in other small tree species [34,35].

### 3.2. Trace Elements Analysis in Leaves of Experimentally Cultivated Germplasm

Trace elements analysis was performed on the leaves of cultivated germplasm without any fertilization treatment to assess the different genotypes. In general, the concentration of essential elements in various plant organs is determined by both intrinsic (genetic) and extrinsic factors (edaphic and climatic) [23,24]. According to the FAO/WHO (1996), microelements are categorized as essential (Cu, Cr, Fe, Mo, Se, and Zn) and probably essential (B, Co, Mn, Ni, Si, Sr, and V) trace elements, which are indispensable for maintaining the healthy function of the body, and potentially toxic elements (Al, As, Be, Cd, Pb, Sn, Sb, and Hg), which can be harmful to the body. In our study, statistically significant differences were observed among genotypes concerning each element. K was the most abundant macro-element in all genotypes, followed by Ca and P, which is in accordance with the relevant literature [36,37]. As far as the essential microelements are concerned, the most abundant was Fe, followed by Zn, Cu, and Cr. Finally, two toxic elements were detected, (As and Pb), but at contents significantly lower than the values allowed by the WHO in herbal substances [38]. As all samples were cultivated in the same pilot field under the same environmental conditions and with no external fertilization (only plants of the control treatment considered), thus eliminating the effect of extrinsic factors on mineral production/accumulation, the observed differences could be attributed only to genetic factors, thus highlighting the genetic variation between the studied samples.

### 3.3. Fruit Phytochemical Profile, Leaf Total Phenolic Content, and Antioxidant Activity in Conventionally and Organically Cultivated Germplasm

Concerning the effect of fertilization treatment on the physicochemical and phytochemical attributes of *S. nigra* fruits, the results of this work showed that genotype was the decisive factor determining the attributes analysed, as most differences were observed among genotypes within each fertilization treatment when compared with the differences between fertilization treatments within each population. This trend has also been reported in the literature [31]. A strong genotype effect of the current germplasm has also been demonstrated regarding other domestication attributes that were previously evaluated, such as asexual propagation, field establishment, and juvenile tree growth potential [30,39]. Nevertheless, via the current approach, particular germplasm accessions with high phytochemical potential under organic fertilization could be discerned, such as genotype GR-1-BBGK-19,574. Organic and conventional fertilization treatments were applied in the long-term cultivation trial presented herein (still ongoing), presenting a diversified and sustainable cropping system as opposed to traditional, conventionally managed systems [35,40,41,42].

The phenolic content of ripe elderberries cultivated in the same field seemed to be primarily controlled by genotype. The results ranged from 137.56 ± 5.21 mg GAE 100 g^−1^ FW for the conventionally fertilized GR-1-BBGK-19,479 genotype to 375.45 ± 19.16 mg GAE 100 g^−1^ FW for the organically fertilized GR-1-BBGK-19,574 genotype. The antioxidant activity also proven to be genotype-dependent, rather than treatment-dependent, and in most cases, it was particularly high (>70% in seven of the nine assessed genotypes under organic fertilization). The radical scavenging activity of polyphenols is well known, and a high TPC is usually correlated with strong antioxidant activity [20,33]. Previous data in the literature concerning the TPC of ripe elderberries [20,43] report values ranging from 364–582 mg GAE 100 g^−1^ FW to 4917–8974 mg 100 g^−1^ DW. Various studies employing different assays [2,5,33,43] also confirm the potent antioxidant activity of *S. nigra* fruits.

Concerning further phytochemical parameters that were measured in fruits, such as TF and ascorbic acid, the examined genotypes presented significant differences outweighing the effects of fertilization, which were scarce. Specific genotypes could be discerned, such as GR-1-BBGK-19,637 and GR-1-BBGK-19,574, showing high ascorbic acid and TF contents compared with other genotypes, yet without significant differences between fertilization treatments. Similar studies have outlined the effects of the genetic factor among *S. nigra* genotypes in terms of fruit phytochemical profile [31]. Additional studies on the Greek native germplasm of other shrub species, such as *Rosa canina*, have shown strong genotypic variability in the TPC, AA, TF, and ascorbic acid of fruits [26]. The ascorbic acid content of fruits has been shown to be positively correlated with TSS (°Brix) (which was also measured herein), and the TF content of fruits has been shown to be positively correlated with TPC in the wild germplasm of other small tree species, such as *Cornus mas* [29]. Lastly, pH and TA (g citric acid/100 g) could indicate a fruit’s maturity status, but differences among genotypes were only observed herein for TA. The maturity index of fruits, expressed as the ratio of the sugar content (°Brix) to the malic acid content (g MA/100 g), was not found to be correlated with AA, TPC, TF and ascorbic acid in cornelian cherries [29]. In any case, further research on the relationship between the fruit maturity status and the phytochemical profile of *S. nigra* fruits is needed.

The leaves also showed remarkable antiradical activity, even stronger than the respective AA of fruits (>90%), but with no statistically significant differences between genotypes and fertilization treatments. This trend was accompanied by higher contents of polyphenols, ranging from 165.85 ± 47.75 mg GAE 100 g^−1^ DW for the conventionally fertilized GR-1-BBGK-19,562 genotype to 494.31 ± 44.23 mg GAE 100 g^−1^ DW for the conventionally fertilized GR-1-BBGK-19,596 genotype, with statistically significant differences observed only between genotypes under the same treatment. As in the case of fruits, these results also indicate a strong genetic variation between the assessed germplasm materials, which is also in agreement with the ^1^H-NMR spectra results of leaf extracts and the concomitant decisive role in determining the phenolic profile of the analysed samples. Our results are in accordance with those of other studies in the literature, because, in most of them, elder leaves tended to be richer in phenolic compounds, exhibiting stronger antioxidant activity compared with the fruits [4,44]. Even though the *S. nigra* individuals assessed herein were in their early stage, the current results can provide initial information about the phytochemical potential of the studied germplasm during its first years, thus creating a basis for further evaluation as plants grow into maturity over the years (ongoing field trial).

## 4. Materials and Methods

### 4.1. Plant Material

**Figure 2:**
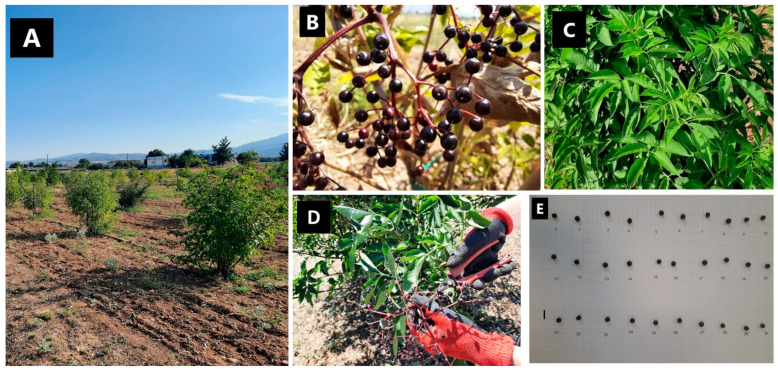
Representative photos of the materials used for phytochemical and physicochemical analyses from cultivated germplasm of *Sambucus nigra* genotype GR-1-BBGK-19,73. (**A**) Two-year-old *S. nigra* plants established at the cultivation trial; (**B**) ripe *S. nigra* fruit clusters; (**C**) composite leaves taken for analysis; (**D**) collection of fruits; and (**E**) fruit examination before analysis (inside bar indicates 1 cm).

The plant material used in this study originated from ex situ experimentally cultivated germplasm of nine Greek *S. nigra* genotypes. These population samples were previously documented and molecularly authenticated directly from the wild, and were assigned a unique IPEN (International Plant Exchange Network) accession number given by the Balkan Botanic Garden of Kroussia (BBGK) of the Institute of Plant Breeding and Genetic Resources (IPBGR), Hellenic Agricultural Organization Demeter in Thermi, metropolitan Thessaloniki, Greece (IPBGR, ELGO-Dimitra), as described in Karapatzak et al. [30]. These *S. nigra* genotypes were established in a pilot field trial at the premises of BBGK-IPBGR in March 2020 using individuals that were produced via cutting propagation, thus securing the uniformity of the material from each original population [30] (Figure 2). The IPEN accession numbers of the nine genotypes used in this study were as follows: GR-1-BBGK-19,73; GR-1-BBGK-19,192; GR-1-BBGK-19,425; GR-1-BBGK-19,479; GR-1-BBGK-19,562; GR-1-BBGK-19,574; GR-1-BBGK-19,596; GR-1-BBGK-19,629; and GR-1-BBGK-19,637.

### 4.2. Total Phenolic Content and Antioxidant Activity of Fruits in Wild-Growing and Cultivated Germplasm

Ripe fruits were collected during targeted botanical expeditions in 2021 from wild-growing individuals, namely from two genotypes among those cultivated and studied herein (Supplementary Table S1). The collected fruits from the wild were visually free of pest infestation or disease infection. It must be noted that, for most of the investigated wild-growing populations, ripe fruits were extremely difficult to locate, as they were collectively consumed by wild bird populations. Fruit ripeness was visually assessed in terms of colour (fully ripe fruits were shiny black–violet) and via fruit texture (fully ripe fruits started to soften). From each wild population, fully ripe fruit clusters were collected from wild-growing individuals during July 2021. The number of sampled individuals varied depending on the local occurrence of individuals in each locality (3–5 individuals). Similarly, the number of sampled fruit clusters from each wild individual tree also varied depending on maturity status (3–10 clusters per individual). Only mature fruits were collected. The collections resulted in a composite sample from each population and locality of 30–50 fully ripe fruits, which were moved to the laboratory for analysis. The two population samples that were used to compare the TPC and AA between wild-growing and cultivated germplasm were GR-1-BBGK-19,425 and GR-1-BBGK-19,629. Detailed protocols used for fruit collection from cultivated germplasm are described in Karapatzak et al. [29].

The ex situ field cultivation trial took place in the grounds of the BBGK-IPBGR in Thermi, metropolitan Thessaloniki, Greece (40.534934 N, 23.002401 E, 40 m elevation). Details of the environmental and soil conditions of the pilot field are described in Karapatzak et al. [35], where a cultivation trial in the same location was conducted on Greek germplasm of various forest species, including *S. nigra*, *Rosa canina* L., and *Amelanchier ovalis* Medik., among others. Three fertilization treatments were applied: (a) conventional crop fertilization rich in nitrogen, (b) organic crop fertilization using organic fertilizers containing organic acids, amino acids, humic acid, and nitrogen, and (c) no fertilization (control). The fertilization regimes were empirically designed and applied gradually over each growing season. The applied fertilization regimes have also been used previously in a cultivation trial of Greek germplasm of different species such as *A. ovalis*; thus, a detailed description of the fertilization regimes applied is provided in Karapatzak et al. [35] and Supplementary Table S1 therein. The field experiment resulted in 27 treatments in total under a complete randomized design, with five replicate plants per treatment [30].

#### 4.2.1. Preparation of Fruit Extracts

The elderberry fruit extracts were prepared according to the method described by Vavoura et al. [45], with some modifications as follows: part of the homogenized fruit sample (2–5 g) was mixed with 10 mL of MeOH_(aq)_ 60% (*v*/*v*), and the mixture was centrifuged (Biofuge primo R, Heraeus, Thermo Fisher Scientific, Waltham, MA, USA) at 4 °C and 4000 rpm. The supernatant was collected, the volume was made up to 20 mL, and this extract was used for the analysis of the total phenolic content (TPC) and antioxidant activity (AA) of the fruits. The precise methods for the determination of TPC and AA are described in detail below.

#### 4.2.2. Determination of Total Phenolic Content (TPC) of Fruits

The TPC of the fruit extracts, as described above, was measured using the method described by Vavoura et al. [45]. In brief, 0.20 mL of fruit extract along with 2.3 mL of deionized H_2_O and 0.25 mL of Folin–Ciocalteu reagent were added to a volumetric flask. After 3 min, 0.50 mL of Na_2_CO_3_ 20% was added, and the volume was made up to 5 mL. The solution was stored in a dark place for 2 h. Consequently, the absorbance was measured at 725 nm against a blank solution using an Infinite M Nano spectrophotometer (Tecan, Männedorf, Switzerland). The total phenolics were calculated using a gallic acid standard curve (0–0.2 mg mL^−1^, R^2^ = 0.999). The results are expressed as gallic acid equivalents per 100 grams of fresh weight (mg GAE 100 g^−1^ FW). The assay was performed in triplicate and the results represent the mean of three replicates ± SD for each sample.

#### 4.2.3. Determination of Antioxidant Activity (AA) of Fruits

The determination of the antioxidant activity using the prepared fruit extracts was performed according to a modification of a method by Fattahi et al. [46]. In brief, 0.1 mL of fruit extract along with 2.9 mL of DPPH (0.10 mM in MeOH) were added to a 5 mL plastic cuvette, which was stored in the dark for 15 min. The mixture’s absorbance was measured at 517 nm using an Infinite M Nano spectrophotometer (Tecan, Männedorf, Switzerland). The results were expressed as the percentage of radical scavenging activity (% RSA) calculated with the following formula:

% RSA = [(Ao − As)/Ao] × 100

where Ao = absorbance of control and As = absorbance of the sample.

The assay was performed in triplicate and the results represent the mean of three replicates ± SD for each sample.

### 4.3. Fruit Phytochemical Profile of Cultivated Greek Germplasm

The phytochemical profile of fruits from the conventionally and organically cultivated germplasm of nine population samples was assessed via the total phenolic content (TPC) and antioxidant activity (AA), as described above, and it was further assessed via total flavonoids (TF) coupled with the vitamin C content and the physicochemical attributes of total soluble solids (TSS, °Brix), total acidity (TA), and pH. Fruits were collected from the plants of the field trial (Figure 2). Fruit collection was conducted recurrently over a two-month period in 2021 following the evaluation of their ripeness status after visual examination based on colour and berry texture. Fruit clusters were collected separately from each treatment and each replicate plant per treatment. Immediately after collection, the fruits were stored at −18 °C until all ripe fruit clusters were collected per treatment, which were stored separately for each replicate plant. The number of collected fruit clusters per plant varied (5–>100). Consecutively, from each replicate plant, a composite sample of 30 fruits was randomly collected for phytochemical analysis. The preparation of fruit extracts was conducted as described above.

#### 4.3.1. Determination of Total Flavonoid Content (TFC) of Fruits

The total flavonoid content in fruits was determined according to the protocol of Goud and Prasad [22], with some modifications, as follows: firstly, 0.5 mL of fruit extract was added to 2 mL distilled water. Then, 0.15 mL of NaNO_2_ 5% was added, followed by mixing, and the reaction mixture was stored in the dark for 5 min. Afterwards, 0.15 mL of 10% AlCl_3_.6H_2_O was added and mixed. After a second incubation in the dark (6 min at room temperature), 1 mL of 1 M NaOH was added, and the volume was made up to 5 mL with distilled water. The absorbance was measured at 510 nm against H_2_O as a blank using an Infinite M Nano spectrophotometer (Tecan, Männedorf, Switzerland). The total flavonoids were calculated using a catechin standard curve (0–0.06 mg mL^−1^, R^2^ = 0.998), and the results were expressed as catechin equivalents per 100 grams of fresh weight (mg CE 100 g^−1^ FW). The assay was performed in triplicate, and the results represent the mean of three replicates ± SD for each sample.

#### 4.3.2. Determination of Ascorbic Acid Content of Fruits

The determination of ascorbic acid in fruits was carried out according to the protocol of Lee and Coates [47], with some modifications, using an HPLC system (Agilent, model 1100 series, Agilent Co., Santa Clara, CA, USA). In brief, 2–5 g of homogenized fruit sample was added to a centrifuge tube with 5 mL of 4.5% *w*/*v* metaphosphoric acid (MPA) solution, and the mixture was stirred and centrifuged (8000 rpm, 4 °C) for 20 min. Then, 1 mL of the supernatant was collected and diluted up to 10 mL with 4.5% MPA solution. The above-mentioned solution was filtered through 0.45 μm polyethersulfone filters and the filtrate was stored at 4 °C until HPLC-DAD analysis, covered with aluminium foil to prevent the oxidation of ascorbic acid. The HPLC-DAD conditions were as follows: Column (Agilent Eclipse XDB-C18) 4.6 mm × 150 mm, 5 μm; elution solvent: 0.005 M aqueous H_2_SO_4_ solution, isocratic elution, flow rate of 0.5 mL min^−1^, and detector wavelength 245 nm. Ascorbic acid was calculated using an ascorbic acid standard curve (0–0.005 mg mL^−1^, R^2^ = 0.997). The results were expressed in milligrams of ascorbic acid per 100 g of fresh weight (mg ascorbic acid 100 g^−1^ FW), calculated from the mean of three replicates ± SD for each sample.

#### 4.3.3. Total Soluble Solids (TSS, °Brix), Total Acidity (TA), and pH

The pH of *S. nigra* fruits was measured using a pH meter (Delta OHM, HD 345, Selvazzano Dentro, Italy). The total soluble solids content (TSS) was determined through Brix degree (°Brix) measurement using a refractometer (Hanna Instruments, refractometer RB 32, Leighton Buzzard, UK). For the titratable acidity, part of the homogenized fruit samples (1–2 g) was mixed with distilled water, and the mixture was centrifuged (Biofuge primo R, Heraeus, Thermo Fisher Scientific, Waltham, MA, USA) at 4 °C and 4000 rpm. The supernatant was collected, and the titratable acidity (TA) was measured by titrimetry using 0.1 M NaOH until the colour changed. The pH of the supernatant was measured at the endpoint of titration (pH = 8.5). The results were expressed in grams of citric acid per 100 grams of fresh weight, calculated from the mean of three measurements ± SD for each sample.

### 4.4. Total Phenolic Content and Antioxidant Activity of Leaves of Cultivated Germplasm

#### 4.4.1. Preparation of Leaf Extracts

Leaf samples from all cultivated plants were collected during July 2020 (Figure 2). Uniformity during leaf collection was based on leaf age and internode position. The collected leaves were obtained from the middle internodes of fully developed lateral shoots, thus representing the main photosynthetically active leaves of the plant. Samples were taken from each of the five plants per treatment, resulting in a composite sample of 50 ± 5 g dry weight from each replicate plant per treatment. The samples were firstly air-dried in the dark at room temperature and subsequently comminuted using a grinding mill (IKA A 10 basic). Dried and comminuted *S. nigra* leaves were extracted with 70% aqueous methanol (MeOH_(aq)_) at a ratio of 1:30 (1 g of dried plant tissue to 30 mL of extraction solution) using a sonicator bath for 10 min (room temperature). The extract was filtered through a paper filter and the sediment was re-extracted for a second time with 30 mL of MeOH_(aq)_; the two filtrates were combined and then dried in vacuo (40 °C, Rotavapor^®^ R-210, Büchi, Labortechnik, Flawil, Switzerland). ^1^H-NMR (nuclear magnetic resonance) spectra of leaf extracts were recorded using an AGILENT DD2 500 NMR spectrometer (20 mg of extract dissolved in 500 µL of CD_3_OD) operating at 500 MHz. Chemical shifts were reported in ppm (*δ*). For the in vitro determination of TPC and AA, the extracts were reconstituted with DMSO at a concentration of 10 mg mL^−1^.

#### 4.4.2. Determination of Total Phenolic Content (TPC) of Leaves

The TPC of *S. nigra* leaves was determined by means of the Folin–Ciocalteu method [35]. In brief, 0.02 mL of extract was mixed with 2.5 mL of deionized H_2_O and 0.40 mL of Folin–Ciocalteu reagent (F9252, Sigma-Aldrich, Darmstadt, Germany) and left to stand in the dark for 8 min at room temperature. An amount of 0.50 mL of Na_2_CO_3_ 7% (*w*/*v*) was subsequently added and the reaction mixture was incubated for 30 min in the dark at 40 °C. The samples’ absorbance was measured at 750 nm against a blank solution using a UV-vis spectrophotometer (UV-1700 PharmaSpec, Shimadzu, Kyoto, Japan), and their TPC was calculated with the aid of a gallic acid standard curve (0–1.5 mg mL^−1^, R^2^ = 0.947). The results were expressed in milligrams of gallic acid equivalents per 100 grams of dry weight (mg GAE 100 g^−1^ DW). The assay was performed in triplicate and the results represent the mean of three replicates ± SD for each sample.

#### 4.4.3. Evaluation of Antioxidant Activity (AA) of Leaves

The extracts’ antioxidant activity was evaluated using the DPPH (2,2-diphenyl-1-picrylhydrazyl) method following the protocol of Karapatzak et al. [35]. In brief, an aliquot of 1.98 mL of DPPH (D211400, Sigma-Aldrich, Darmstadt, Germany) solution in MeOH (0.1 mM) was added to 0.02 mL of extract, and the mixture was incubated in the dark for 20 min at room temperature. The absorbance of the reaction mixture was read at 517 nm using a UV-vis spectrophotometer (UV-1700 PharmaSpec, Shimadzu, Kyoto, Japan). The results were expressed as the percentage of radical scavenging activity (% RSA), calculated using the same formula as that in Section 4.2.3. The assay was performed in triplicate and the results represent the mean of three replicates ± SD for each sample.

### 4.5. Trace Elements Analysis in Leaves of Cultivated Germplasm

Leaf samples from the cultivated control treatments of each genotype of *S. nigra* were collected as described in Section 4.4.1 and were subjected to ICP-OES analysis to determine the content of 24 minerals in the *S. nigra* leaves. For the preparation of the samples in liquid form, the microwave digestion technique was employed. In brief, 2.0 g of dried plant material was dissolved in 65% of HNO_3_, then heated for about 1 h at 120 °C to achieve complete dissolution of the samples. In the final phase of heating, small volumes (2–3 mL) of hydrogen peroxide were added. After filtering the samples, the volume was adjusted to 25 mL for each extract. For the elemental analysis, an iCAP 6000 inductively coupled plasma–optical emission spectrometer (Thermo Scientific, Cambridge, UK) with an echelle optical design and a charge injection device (CID) solid-state detector was used under the following operating conditions: flush pump rate: 100 rpm; analysis pump rate: 50 rpm; RF power: 1150 W; nebulizer gas flow: 0.7 L min^−1^; coolant gas flow: 12 L min^−1^; auxiliary gas flow: 0.5 L min^−1^; and plasma view axial time of rinse: 30 s. The multielement standard solution III of macro-elements, i.e., calcium (Ca), potassium (K), magnesium (Mg), phosphorus (P), and sodium (Na) (TraceCert, Fluka Analytical, Buchs, Switzerland), multi-element standard solution IV of microelements, i.e., aluminium (Al), arsenic (As), boron (B), barium (Ba), cobalt (Co), chromium (Cr), copper (Cu), iron (Fe), manganese (Mn), nickel (Ni), lead (Pb), silicon (Si), and zinc (Zn) (TraceCert, Fluka Analytical, Switzerland), and single-element standard solutions of Hg, Si, and P (TraceCert, Fluka Analytical, Buchs, Switzerland) were used for calibration. All measurements were performed in triplicate and the results were expressed in mg g^−1^ DW (for macro-elements) or μg g^−1^ DW (for microelements) ± SD.

### 4.6. Statistical Analysis

For the comparison of fruit TPC and AA between wild-growing and cultivated germplasm, a GLM ANOVA was applied to the data values that were measured in triplicate (*n* = 3) to determine the effects of germplasm type and population sample (genotype) on the variables measured. In addition, mean comparison was conducted via Tukey’s HSD post hoc test (*p* < 0.05) discreetly among genotypes for each germplasm type and among germplasm types for each genotype. Similarly, for leaf trace element analysis, an analysis of variance (*n* = 3) was applied to explore differences among population samples (genotypes) for each element measurement separately, whereas mean comparison was conducted via Tukey’s HSD post hoc test (*p* < 0.05).

To avoid potential pseudo-replication issues for the comparison of the effects of conventionally vs. organically cultivated germplasm on the variables measured (fruit and leaf TPC and AA, as well as fruit phytochemical and physicochemical attributes), the average values of each analysis that was conducted in triplicate for each replicate plant were used in the analysis, which followed the field trial design described above, having each replicate plant as the experimental unit (*n* = 5). A GLM ANOVA was applied to establish the effects of fertilization regime and population sample (genotype) on the variables measured, whereas mean comparisons were conducted via Tukey’s HSD post hoc test (*p* < 0.05) discreetly among genotypes for each fertilization regime and among fertilization regimes for each genotype. All analyses were conducted using IBM-SPSS 23.0 software.

## 5. Conclusions

The establishment of Greek *S. nigra* germplasm in a pilot cultivation system, screening the different genotypes under field conditions, sets the basis for its domestication and sustainable utilization. This effort has already yielded early results regarding agronomical aspects. Thus, this study provides novel data on the phytochemical profile of experimentally cultivated Greek *S. nigra* germplasm and adds to the work previously conducted regarding the creation of a foundational framework for the sustainable agronomic exploitation of Greek *S. nigra*. The approach applied herein outlines the high phytochemical and antioxidant potential of selected Greek *S. nigra* genotypes, coupled with its diversity among different population samples, thus facilitating targeted genotype selection for future research and breeding efforts, phenotyping, and genetic improvement of any prioritized genotypes. Therefore, this study makes an important towards the sustainable exploitation of this species, with high pharmaceutical and nutritional value.

## Figures and Tables

**Table 1:** Total phenolic content (TPC, mg GAE 100 g^−1^ ± SD) and antioxidant activity (AA, % RSA ± SD, *n* = 3) in fruit samples of wild-growing and cultivated germplasm of two Greek *Sambucus nigra* genotypes.

Genotype	Cultivated		Wild-Growing	
	TPC ± SD	AA ± SD	TPC ± SD	AA ± SD
GR-1-BBGK-19,425	306.37 ± 54.80 a	88.05 ± 9.31	97.09 ± 0.02 b	90.49 ± 0.35
GR-1-BBGK-19,629	225.83 ± 67.40 a	74.66 ± 9.08	86.75 ± 0.01 b	81.91 ± 0.55

Significant differences were only observed in TPC between cultivated and wild-growing germplasm and are denoted with different letters based on Tukey’s HSD (*p* < 0.05).

**Table 2:** Concentrations of macro-elements measured in leaves of cultivated germplasm of nine Greek *Sambucus nigra* genotypes (mg g^−1^ DW ± SD, *n* = 3).

Genotype	Ca	K	Mg	Na	P
GR-1-BBGK-19,192	6628.975 ± 43.541 c	5972.277 ± 57.193 a	2421.807 ± 18.066 e	1.688 ± 0.018 f	2444.951 ± 15.235 c
GR-1-BBGK-19,425	5564.999 ± 60.142 b	10,053.423 ± 239.941 f	2065.322 ± 20.280 c	0.722 ± 0.022 c	2334.871 ± 6.760 c
GR-1-BBGK-19,479	3675.253 ± 71.763 a	7550.155 ± 59.651 bc	2278.113 ± 6.005 d	0.820 ± 0.017 d	3240.244 ± 27.366 e
GR-1-BBGK-19,562	6801.939 ± 632.317 c	6850.740 ± 711.519 b	1887.522 ± 54.015 a	0.609 ± 0.072 b	2187.187 ± 75.390 b
GR-1-BBGK-19,574	3282.890 ± 17.024 a	8895.135 ± 168.102 de	2063.519 ± 7.864 c	1.116 ± 0.024 e	3311.785 ± 116.197 e
GR-1-BBGK-19,596	3677.508 ± 78.599 a	9500.091 ± 102.533 ef	2071.192 ± 9.178 c	1.040 ± 0.011 e	2453.885 ± 4.002 c
GR-1-BBGK-19,629	3181.210 ± 21.599 a	8252.812 ± 167.667 cd	1950.266 ± 6.822 b	0.526 ± 0.018 ab	2368.564 ± 4.291 c
GR-1-BBGK-19,637	3062.251 ± 61.281 a	6875.235 ± 98.955 b	1935.948 ± 12.293 ab	0.515 ± 0.011 a	1896.263 ± 2.454 a
GR-1-BBGK-19,73	3619.089 ± 28.115 a	7016.971 ± 74.622 b	2088.369 ± 8.575 c	1.603 ± 0.022 f	3062.647 ± 10.280 d

Means that do not share the same letter within each element column are significantly different (Tukey’s HSD, *p* < 0.05). All analyses were conducted in triplicate.

**Table 3:** Concentrations of micro-elements measured in leaves of cultivated germplasm of nine Greek *Sambucus nigra* genotypes (μg g^−1^ DW ± SD, *n* = 3).

Genotype	Al	As	B	Ba	Co	Cr	Cu	Fe	Mn	Ni	Pb	Si	Zn
GR-1-BBGK-19,192	213.871 ± 0.968 f	0.343 ± 0.063 bcd	75.262 ± 0.501 f	39.620 ± 0.694 d	0.344 ± 0.006 d	0.881 ± 0.036 e	4.563 ± 0.012 d	211.391 ± 2.272 e	151.755 ± 0.429 f	1.896 ± 0.007 e	0.554 ± 0.012 bc	7.824 ± 0.031 e	14.663 ± 0.046 e
GR-1-BBGK-19,425	363.728 ± 1.483 g	0.501 ± 0.011 d	38.861 ± 0.184 b	22.988 ± 0.244 c	0.509 ± 0.011 e	1.756 ± 0.049 f	4.490 ± 0.032 d	342.642 ± 3.648 f	101.258 ± 0.724 e	3.311 ± 0.009 g	0.816 ± 0.030 e	2.109 ± 0.017 a	14.711 ± 0.066 e
GR-1-BBGK-19,479	124.983 ± 1.765 b	0.206 ± 0.027 abc	44.077 ± 0.426 c	15.046 ± 0.167 a	0.276 ± 0.012 bc	0.549 ± 0.042 bcd	4.163 ± 0.062 c	134.844 ± 2.232 b	87.707 ± 0.627 d	1.334 ± 0.042 b	0.760 ± 0.074 de	7.006 ± 0.104 d	11.720 ± 0.125 a
GR-1-BBGK-19,562	71.939 ± 5.931 a	0.161 ± 0.024 a	35.112 ± 2.385 a	19.754 ± 2.073 b	0.208 ± 0.019 a	0.200 ± 0.054 a	3.630 ± 0.294 b	66.801 ± 4.395 a	74.266 ± 5.019 b	1.086 ± 0.034 a	0.253 ± 0.061 a	2.172 ± 0.180 a	14.475 ± 0.351 e
GR-1-BBGK-19,574	174.237 ± 1.181 e	0.364 ± 0.041 cd	41.963 ± 1.390 c	18.038 ± 0.218 b	0.344 ± 0.003 d	0.693 ± 0.136 d	4.621 ± 0.101 d	167.261 ± 4.634 d	102.193 ± 0.799 e	2.164 ± 0.029 f	0.576 ± 0.065 c	11.930 ± 0.240 f	13.039 ± 0.032 c
GR-1-BBGK-19,596	133.444 ± 1.253 c	0.239 ± 0.069 abc	55.685 ± 0.541 e	19.740 ± 0.174 b	0.295 ± 0.006 c	0.395 ± 0.025 b	4.598 ± 0.064 d	131.078 ± 1.271 b	81.257 ± 0.816 c	1.691 ± 0.007 d	0.490 ± 0.079 bc	4.127 ± 0.042 b	15.266 ± 0.043 f
GR-1-BBGK-19,629	169.714 ± 2.716 e	0.237 ± 0.087 abc	38.518 ± 0.353 b	14.180 ± 0.427 a	0.293 ± 0.008 c	0.618 ± 0.037 d	3.327 ± 0.037 b	139.198 ± 3.137 b	55.881 ± 0.618 a	1.710 ± 0.012 d	0.481 ± 0.040 bc	2.054 ± 0.023 a	13.639 ± 0.086 d
GR-1-BBGK-19,637	135.009 ± 0.746 c	0.184 ± 0.045 ab	36.149 ± 0.174 ab	15.084 ± 0.255 a	0.256 ± 0.010 b	0.442 ± 0.005 bc	2.918 ± 0.021 a	134.804 ± 2.155 b	97.447 ± 1.881 e	1.513 ± 0.006 c	0.393 ± 0.105 ab	6.604 ± 0.140 c	12.519 ± 0.024 b
GR-1-BBGK-19,73	162.419 ± 0.608 d	0.271 ± 0.083 abc	47.428 ± 0.183 d	17.648 ± 0.211 b	0.286 ± 0.001 c	0.564 ± 0.010 cd	4.096 ± 0.008 c	154.482 ± 1.394 c	85.497 ± 0.523 cd	2.149 ± 0.002 f	0.594 ± 0.051 cd	7.319 ± 0.036 d	18.147 ± 0.038 g

Means that do not share the same letter within each element column are significantly different (Tukey’s HSD, *p* < 0.05). All analyses were conducted in triplicate.

**Table 4:** Results of the phytochemical analyses of fruits of nine genotypes of Greek *Sambucus nigra* that were established in the pilot cultivation trial under conventional fertilization, organic fertilization, and control (no fertilization). The measured phytochemical attributes were total flavonoids (TF, mg catechin equivalents 100 g^−1^) and ascorbic acid content (AA, mg ascorbic acid 100 g^−1^), whereas the measured physicochemical attributes were pH, total soluble solids (TSS, °Brix), and total acidity (TA, g citric acid 100 g^−1^). The results are given as means ± SD (*n* = 5).

	Control					Conventional					Organic				
Genotype	pH	TSS	TA	TF	AA	pH	TSS	TA	TF	AA	pH	TSS	TA	TF	AA
GR-1-BBGK-19,192	5.16 ± 0.19)	16.70 ± 3.81 ab	0.45 ± 0.01 a	117.8 ± 1.49 abcd	0.19 ± 0.00	4.99 ± 0.10	15.6 ± 1.47 ab	0.41 ± 0.04 a	59.5 ± 19.73 a	0.21 ± 0.07 a	5.13 ± 0.00	12.80 ± 0.00	0.66 ± 0.00	90.35 ± 0.00	0.19 ± 0.00
GR-1-BBGK-19,425	5.04 ± 0.01	12.25 ± 0.86 a, A	0.82 ± 0.09 ab, A	174.25 ± 33.67 cd	0.97 ± 0.29 ab	5.12 ± 0.17	14.04 ± 0.27 ab, B	0.82 ± 0.05 c, A	135.39 ± 26.29 b	0.69 ± 0.37 a	4.85 ± 0.17	13.08 ± 0.78 ab, AB	1.18 ± 0.13 b, B	150.98 ± 38.96 ab	0.58 ± 0.11 a
GR-1-BBGK-19,479	4.61 ± 0.28	18.40 ± 4.23 b	0.68 ± 0.103 ab	102.71 ± 21.82 abc	0.89 ± 0.35 ab	4.01 ± 0.01	14.2 ± 0.36 ab	0.72 ± 0.06 bc	88.21 ± 25.94 ab	0.66 ± 0.06 a	4.41 ± 0.45	16.12 ± 1.75 b	0.59 ± 0.06 a	81.40 ± 22.55 a	0.42 ± 0.04 a
GR-1-BBGK-19,562	4.84 ± 0.06	12.64 ± 1.12 ab	0.97 ± 0.24 b, AB	134.31 ± 39.46 abcd	0.33 ± 0.12 a	4.81 ± 0.14	12.74 ± 1.23 a	0.72 ± 0.18 bc, A	103.46 ± 30.73 ab	0.48 ± 0.00	4.76 ± 0.13	12.33 ± 0.25 a	1.14 ± 0.10 b, B	123.75 ± 32.85 a	0.29 ± 0.00
GR-1-BBGK-19,574	4.57 ± 0.27	17.05 ± 3.18 ab	0.83 ± 0.02 ab, B	160.98 ± 2.02 bcd	0.96 ± 0.36 ab	4.86 ± 0.13	17.1 ± 5.65 ab	0.56 ± 0.01 abc, A	142.01 ± 25.31 b	0.55 ± 0.50 a	4.83 ± 0.04	22.25 ± 1.76 c	1.16 ± 0.13 b, C	221.95 ± 37.88 b	2.27 ± 1.69 b
GR-1-BBGK-19,596	4.66 ± 0.11	12.70 ± 0.28 ab	0.38 ± 0.04 a	77.98 ± 18.31 ab	0.63 ± 0.57 ab	4.93 ± 0.34	12.8 ± 0.75 a	0.45 ± 0.16 ab	82.41 ± 18.74 ab	0.48 ± 0.16 a	4.33 ± 0.14	13.86 ± 0.30 ab	0.61 ± 0.26 a	125.90 ± 21.98 a	0.73 ± 0.22 a
GR-1-BBGK-19,629	4.74 ± 0.36	17.70 ± 1.60 ab, B	0.72 ± 0.27 ab	143.61 ± 39.46 abcd, B	1.23 ± 0.86 ab	4.82 ± 0.15	13.9 ± 0.94 ab, A	0.53 ± 0.08 abc	60.25 ± 29.83 a, A	0.2 ± 0.01 a	4.83 ± 0.07	13.95 ± 0.07 ab, A	0.65 ± 0.04 a	115.06 ± 2.16 a, AB	1.07 ± 0.09 ab
GR-1-BBGK-19,637	4.93 ± 0.25	15.46 ± 1.59 ab	0.67 ± 0.11 ab	189.34 ± 36.40 d	1.38 ± 0.19 b	4.96 ± 0.29	24.56 ± 12.93 b	0.79 ± 0.15 c	136.41 ± 56.00 b	1.93 ± 0.06 b	4.07 ± 0.00	16.90 ± 0.00	0.61 ± 0.00	172.67 ± 0.00	0.56 ± 0.00
GR-1-BBGK-19,73	4.56 ± 0.30	13.5 ± 1.14 ab	0.68 ± 0.21 ab	75.14 ± 19.78 a	0.36 ± 0.24 ab	4.80 ± 0.30	13.70 ± 3.02 ab	0.54 ± 0.12 abc	53.61 ± 14.66 a	0.16 ± 0.00	4.73 ± 0.30 a	14.55 ± 2.02 ab	0.81 ± 0.12 ab	76.98 ± 43.82 a	0.31 ± 0.15 a

Means that do not share the same letter are significantly different based on respective Tukey’s HSD post hoc tests (*p* < 0.05). Lowercase letters within each column reflect comparisons between genotypes under the same fertilization treatment. Capital letters within each line reflect comparisons between fertilization treatments in the same genotype and attribute. The cases where SD was 0.0 were not included in the post hoc test, as they stemmed from the assessment of one replicate plant that produced fruits. In columns or lines where no respective lettering is given, no significant differences were observed.

**Table 5:** Effect of fertilization regimes (conventional, organic, and control with no fertilization) on total phenolic content (TPC, mg GAE 100 g^−1^ extract ± SD, *n* = 5) and antioxidant activity (AA, % RSA ± SD, *n* = 5) assessed in fruit samples of Greek native germplasm of nine *Sambucus nigra* genotypes.

TPC			
Genotype	Control	Conventional	Organic
GR-1-BBGK-19,73	175.52 ± 71.36 a	141.85 ± 17.40 a	147.15 ± 16.47 a
GR-1-BBGK-19,192	309.85 ± 114.40 a	187.06 ± 47.14 ab	262.10 ± 0.00)
GR-1-BBGK-19,425	306.37 ± 54.80 a	240.10 ± 30.73 bc	345.34 ± 87.35 b
GR-1-BBGK-19,479	223.37 ± 72.12 a	137.56 ± 5.21 a	242.62 ± 43.49 ab
GR-1-BBGK-19,562	284.00 ± 27.62 a, B	147.14 ± 22.71 ab, A	300.16 ± 109.7 ab, B
GR-1-BBGK-19,574	352.60 ± 57.55 a	288.73 ± 19.24 c	375.45 ± 19.16 b
GR-1-BBGK-19,596	192.45 ± 26.79 a	208.56 ± 43.29 abc	305.46 ± 57.82 ab
GR-1-BBGK-19,629	225.83 ± 67.38 a	123.20 ± 24.38 a	234.10 ± 40.16 ab
GR-1-BBGK-19,637	367.36 ± 103.20 a	281.30 ± 86.05 c	313.60 ± 0.00
**AA**			
**Genotype**	**Control**	**Conventional**	**Organic**
GR-1-BBGK-19,73	69.09 ± 17.14 a	58.81 ± 10.29 ab	57.51 ± 22.52 a
GR-1-BBGK-19,192	65.97 ± 19.48 a	49.73 ± 8.37 a	65.70 ± 0.00
GR-1-BBGK-19,425	88.05 ± 9.31 a	91.11 ± 0.23 d	92.04 ± 1.84 b
GR-1-BBGK-19,479	73.76 ± 20.58 a	41.44 ± 9.99 a	74.95 ± 15.88 b
GR-1-BBGK-19,562	76.78 ± 12.23 a	60.66 ± 14.46 abc	76.89 ± 15.59 b
GR-1-BBGK-19,574	97.47 ± 0.55 a	85.50 ± 12.06 cd	71.92 ± 5.90 b
GR-1-BBGK-19,596	86.80 ± 0.66 a	79.51 ± 10.93 bcd	90.40 ± 1.39 b
GR-1-BBGK-19,629	74.66 ± 9.08 a	68.01 ± 9.18 abcd	76.94 ± 2.14 b
GR-1-BBGK-19,637	86.39 ± 7.65 a	83.57 ± 12.34 bcd	85.08 ± 0.00

Means that do not share the same letter are significantly different based on respective Tukey’s HSD post hoc tests (*p* < 0.05). Lowercase letters within each column reflect comparisons between genotypes under the same fertilization treatment. Capital letters within each line reflect comparisons between fertilization treatments in the same genotype. The cases where SD was 0.0 were not included in the post hoc test as they stemmed from the assessment of one replicate plant that produced fruits.

**Table 6:** Effect of fertilization regimes (conventional, organic, and control with no fertilization) on total phenolic content (TPC, mg GAE 100 g^−1^ extract ± SD, *n* = 5) and antioxidant activity (AA, % RSA ± SD, *n* = 5) assessed in leaf samples of Greek native germplasm of nine *Sambucus nigra* genotypes.

TPC			
Genotype	Control	Conventional	Organic
GR-1-BBGK-19,73	224.92 ± 41.06 abc	312.65 ± 131.90 ab	247.46 ± 37.69 a
GR-1-BBGK-19,192	187.13 ± 37.82 a	276.10 ± 77.42 ab	256.83 ± 21.57 a
GR-1-BBGK-19,425	323.84 ± 46.00 cd	355.94 ± 45.26 bc	336.65 ± 5.73 ab
GR-1-BBGK-19,479	309.84 ± 35.28 bcd	326.43 ± 37.05 ab	374.94 ± 67.94 ab
GR-1-BBGK-19,562	209.05 ± 11.47 ab	165.85 ± 47.75 a	194.37 ± 35.51 a
GR-1-BBGK-19,574	262.14 ± 47.24 abc	228.24 ± 81.57 ab	339.83 ± 43.54 ab
GR-1-BBGK-19,596	376.90 ± 75.37 d	494.31 ± 44.23 c	485.17 ± 201.72 b
GR-1-BBGK-19,629	292.76 ± 71.31 abcd	286.64 ± 42.71 ab	280.06 ± 42.22 a
GR-1-BBGK-19,637	254.86 ± 24.66 abc	282.69 ± 44.44 ab	290.13 ± 72.62 a
**AA**			
**Genotype**	**Control**	**Conventional**	**Organic**
GR-1-BBGK-19,73	93.25 ± 1.64	93.80 ± 3.13	93.49 ± 0.61
GR-1-BBGK-19,192	92.11 ± 1.39	93.93 ± 0.41	94.27 ± 1.02
GR-1-BBGK-19,425	93.53 ± 0.62	93.75 ± 0.23	93.43 ± 0.18
GR-1-BBGK-19,479	93.49 ± 0.26	93.43 ± 0.22	92.81 ± 0.48
GR-1-BBGK-19,562	91.74 ± 0.95	92.23 ± 0.78	92.56 ± 0.52
GR-1-BBGK-19,574	93.70 ± 0.09 B	91.42 ± 1.07 A	94.07 ± 0.34 B
GR-1-BBGK-19,596	93.46 ± 0.29	93.98 ± 0.27	93.90 ± 0.95
GR-1-BBGK-19,629	92.84 ± 0.66 A	94.49 ± 0.76 B	93.40 ± 0.41 AB
GR-1-BBGK-19,637	92.27 ± 0.40	92.18 ± 0.08	93.05 ± 0.56

Means that do not share the same letter are significantly different based on respective Tukey’s HSD post hoc tests (*p* < 0.05). Lowercase letters within each column reflect comparisons between genotypes under the same fertilization treatment. Capital letters within each line reflect comparisons between fertilization treatments within the same genotype. Where no lettering is given, no significant differences were observed.

## Data Availability

All data supporting the results of this study are included in the manuscript and/or Supplementary Material, and datasets are available upon request.

## Supplementary Materials

The following supporting information can be downloaded at: https://www.mdpi.com/article/10.3390/plants12081701/s1, Figure S1: ^1^H-NMR spectra of hydromethanolic extracts of *Sambucus nigra* leaves from genotype GR-1-BBGK-19,73 under (a) no fertilization (control), (b) conventional, and (c) organic fertilization treatment (CD_3_OD, 500 MHz). Chemical shifts are reported in ppm on the *x*-axis (0.0–8.0); Figure S2: ^1^H-NMR spectra of hydromethanolic extracts of *Sambucus nigra* leaves from genotype GR-1-BBGK-19,192 under (a) no fertilization (control), (b) conventional, and (c) organic fertilization treatment (CD_3_OD, 500 MHz). Chemical shifts are reported in ppm on the *x*-axis (0.0–8.0); Figure S3: ^1^H-NMR spectra of hydromethanolic extracts of *Sambucus nigra* leaves from genotype GR-1-BBGK-19,425 under (a) no fertilization (control), (b) conventional, and (c) organic fertilization treatment (CD_3_OD, 500 MHz). Chemical shifts are reported in ppm on the *x*-axis (0.0–8.0); Figure S4: ^1^H-NMR spectra of hydromethanolic extracts of *Sambucus nigra* leaves from genotype GR-1-BBGK-19,479 under (a) no fertilization (control), (b) conventional, and (c) organic fertilization treatment (CD_3_OD, 500 MHz). Chemical shifts are reported in ppm on the *x*-axis (0.0–8.0); Figure S5: ^1^H-NMR spectra of hydromethanolic extracts of *Sambucus nigra* leaves from genotype GR-1-BBGK-19,562 under (a) no fertilization (control), (b) conventional, and (c) organic fertilization treatment (CD_3_OD, 500 MHz). Chemical shifts are reported in ppm on the *x*-axis (0.0–8.0); Figure S6: ^1^H-NMR spectra of hydromethanolic extracts of *Sambucus nigra* leaves from genotype GR-1-BBGK-19,574 under (a) no fertilization (control), (b) conventional, and (c) organic fertilization treatment (CD_3_OD, 500 MHz). Chemical shifts are reported in ppm on the *x*-axis (0.0–8.0); Figure S7: ^1^H-NMR spectra of hydromethanolic extracts of *Sambucus nigra* leaves from genotype GR-1-BBGK-19,629 under (a) no fertilization (control), (b) conventional, and (c) organic fertilization treatment (CD_3_OD, 500 MHz). Chemical shifts are reported in ppm on the *x*-axis (0.0–8.0); Figure S8: ^1^H-NMR spectra of hydromethanolic extracts of *Sambucus nigra* leaves from genotype GR-1-BBGK-19,637 under (a) no fertilization (control), (b) conventional, and (c) organic fertilization treatment (CD_3_OD, 500 MHz). Chemical shifts are reported in ppm on the *x*-axis (0.0–8.0); Table S1: Wild-growing genotypes of Greek native *Sambucus nigra* sampled from wild-growing populations of northern and north-central Greece assigned with different IPEN (International Plant Exchange Network) accession numbers.
