# Supplementary material for: Wild-Growing and Conventionally or Organically Cultivated Sambucus nigra Germplasm: Fruit Phytochemical Profile, Total Phenolic Content, Antioxidant Activity, and Leaf Elements
Source: Plants (Basel). 2023 Apr 19;12(8):1701. doi: 10.3390/plants12081701 (PMC10146997; doi:10.3390/plants12081701)
Supplement: Supplementary file 1 [file plants-12-01701-s001.zip › plants-2292606-supplementary.pdf]

Wild-Growing and Conventionally or Organically Cultivated *Sambucus nigra* Germplasm: Fruit Phytochemical Profile, Total Phenolic Content, Antioxidant Activity, and Leaf Elements

Papagrigoriou, T.; Iliadi, P.; Mitić, M.N.; Mrmošanin, J.M.; Papanastasi, K.; Karapatzak, E.; Maloupa, E.; Gkourogianni, A.V.; Badeka, A.V.; Krigas, N.; Lazari, D.

SUPPLEMENTARY MATERIAL

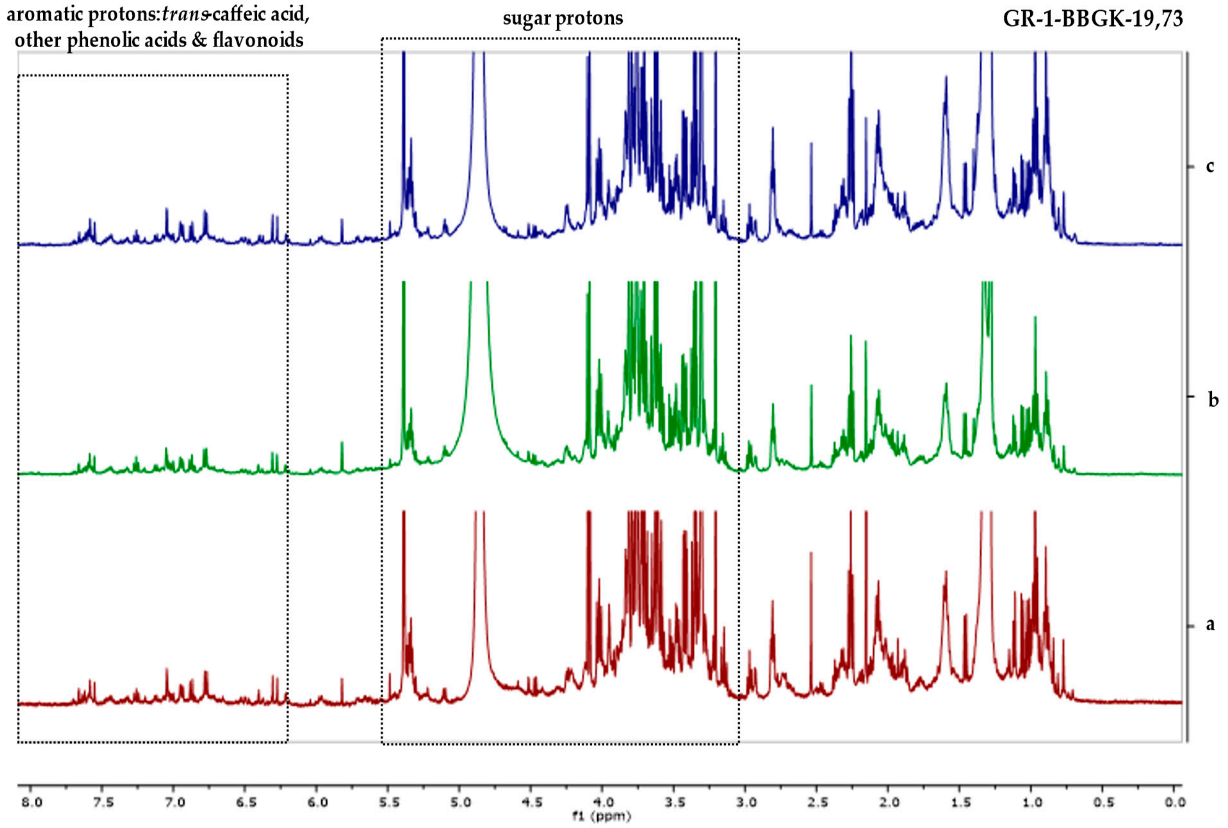

Supplementary Figure S1: ^{1}H-NMR spectra of hydromethanolic extracts of *Sambucus nigra* leaves from genotype GR-1-BBGK-19,73 under (a) no fertilization (control), (b) conventional and (c) organic fertilization treatment (CD_3OD, 500 MHz). Chemical shifts are reported in ppm on the x-axis (0.0 – 8.0).

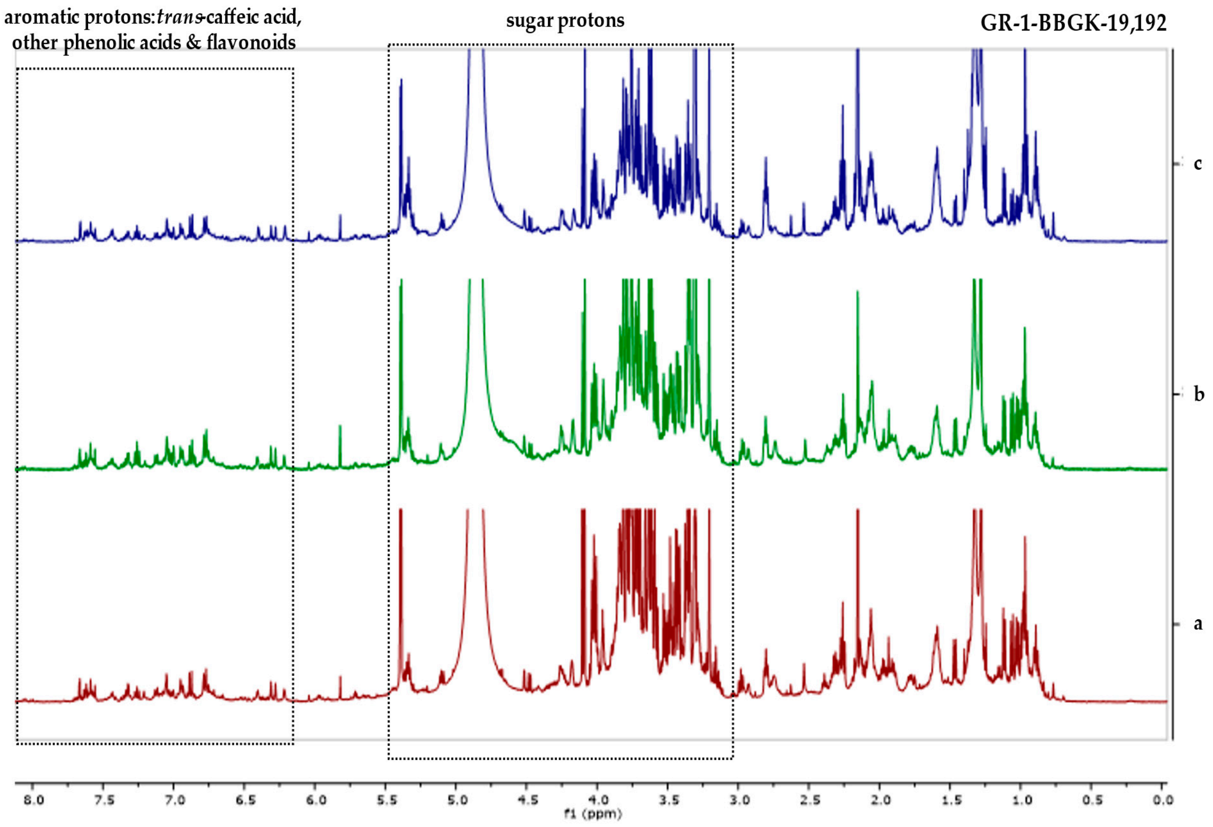

Supplementary Figure S2: ^{1}H-NMR spectra of hydromethanolic extracts of *Sambucus nigra* leaves from genotype GR-1-BBGK-19,192 under (a) no fertilization (control), (b) conventional and (c) organic fertilization treatment (CD3OD, 500 MHz). Chemical shifts are reported in ppm on the x-axis (0.0 – 8.0).

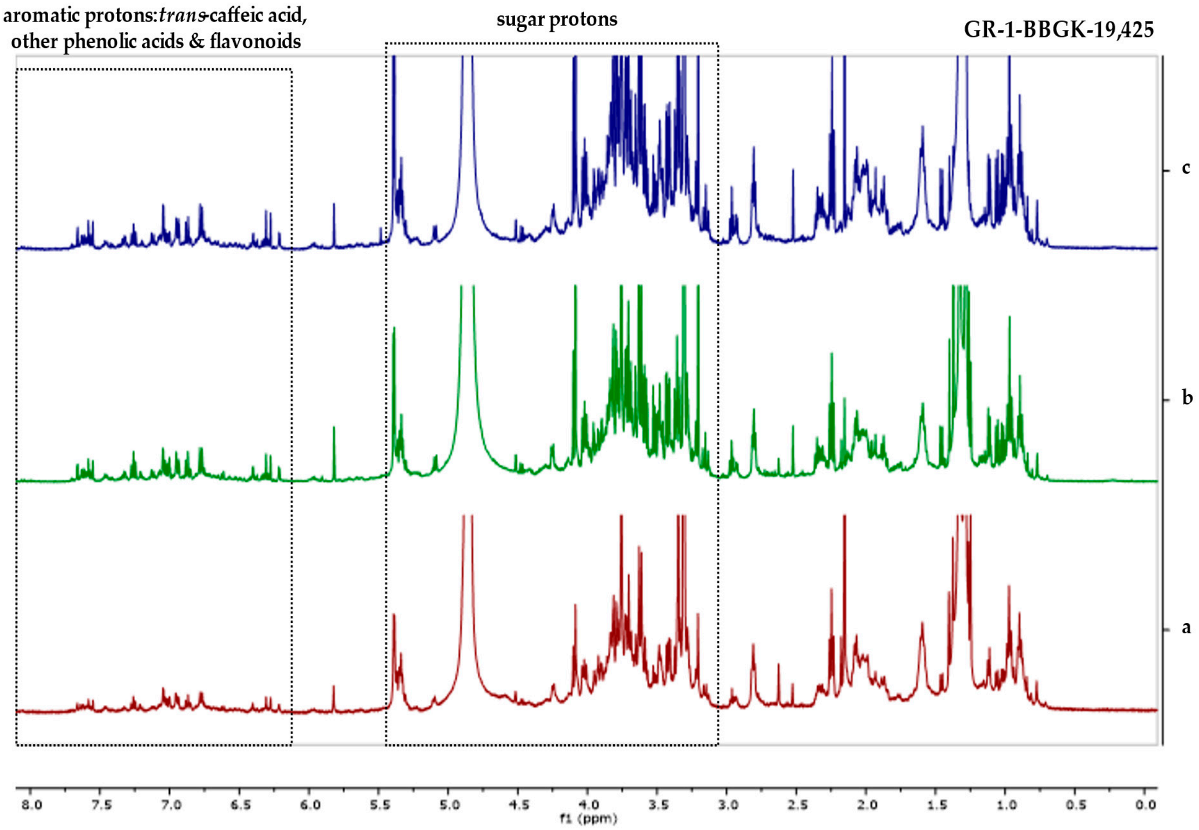

Supplementary Figure S3: ^{1}H-NMR spectra of hydromethanolic extracts of *Sambucus nigra* leaves from genotype GR-1-BBGK-19,425 under (a) no fertilization (control), (b) conventional and (c) organic fertilization treatment (CD3OD, 500 MHz). Chemical shifts are reported in ppm on the x-axis (0.0 – 8.0).

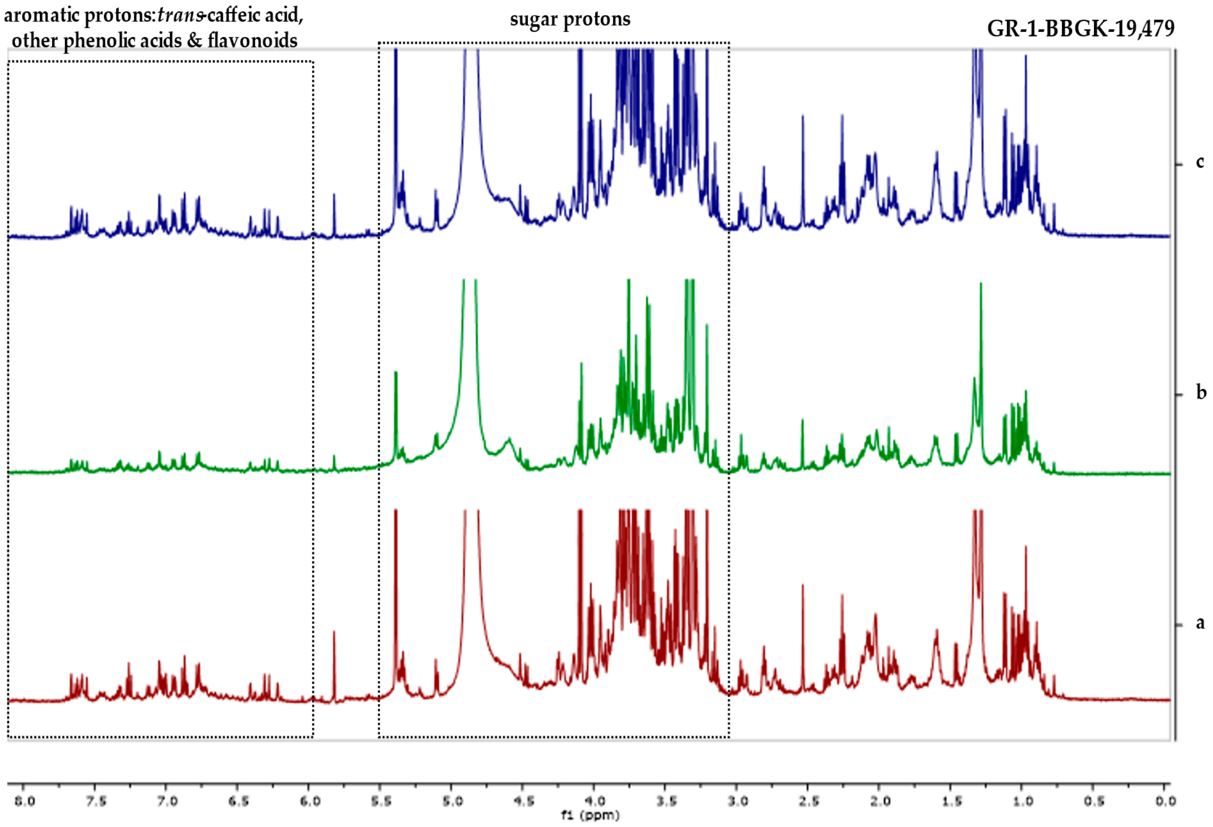

Supplementary Figure S4: ^{1}H-NMR spectra of hydromethanolic extracts of *Sambucus nigra* leaves from genotype GR-1-BBGK-19,479 under (a) no fertilization (control), (b) conventional and (c) organic fertilization treatment (CD3OD, 500 MHz). Chemical shifts are reported in ppm on the x-axis (0.0 – 8.0).

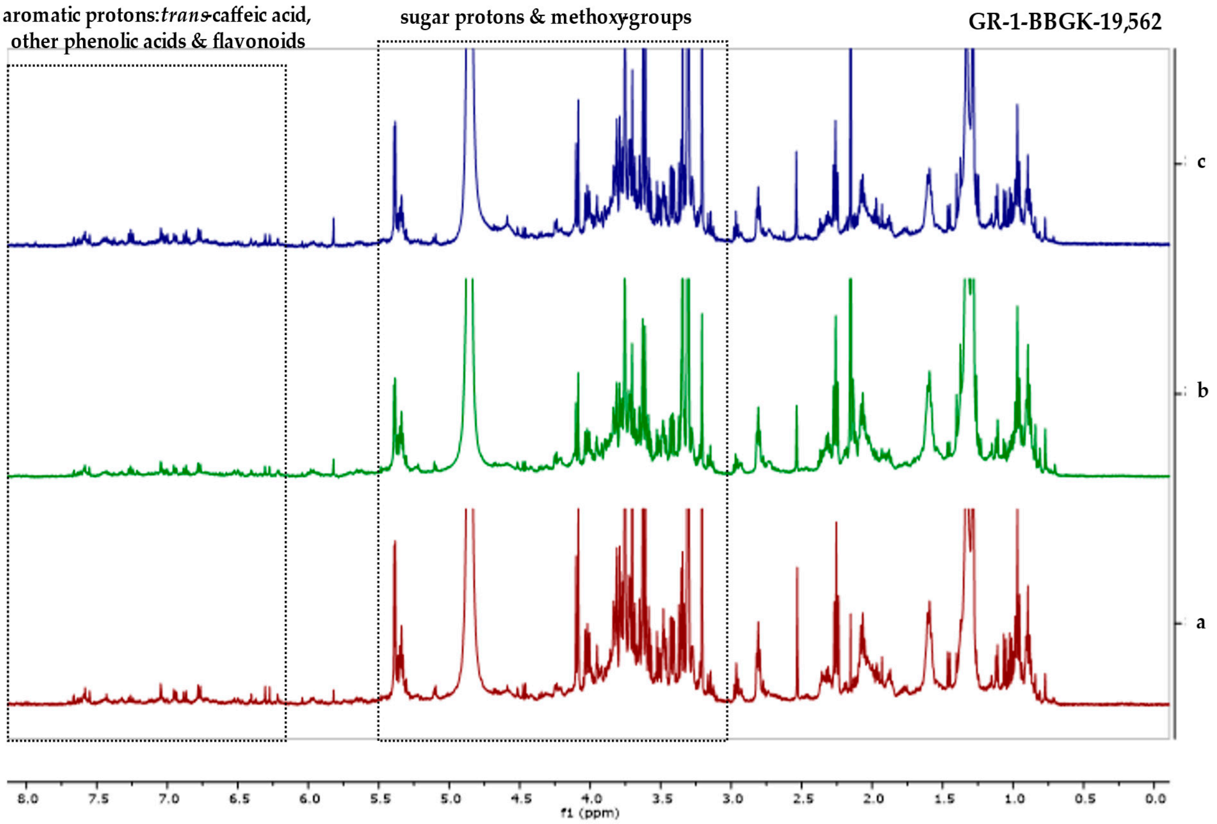

Supplementary Figure S5: ^{1}H-NMR spectra of hydromethanolic extracts of *Sambucus nigra* leaves from genotype GR-1-BBGK-19,562 under (a) no fertilization (control), (b) conventional and (c) organic fertilization treatment (CD3OD, 500 MHz). Chemical shifts are reported in ppm on the x-axis (0.0 – 8.0).

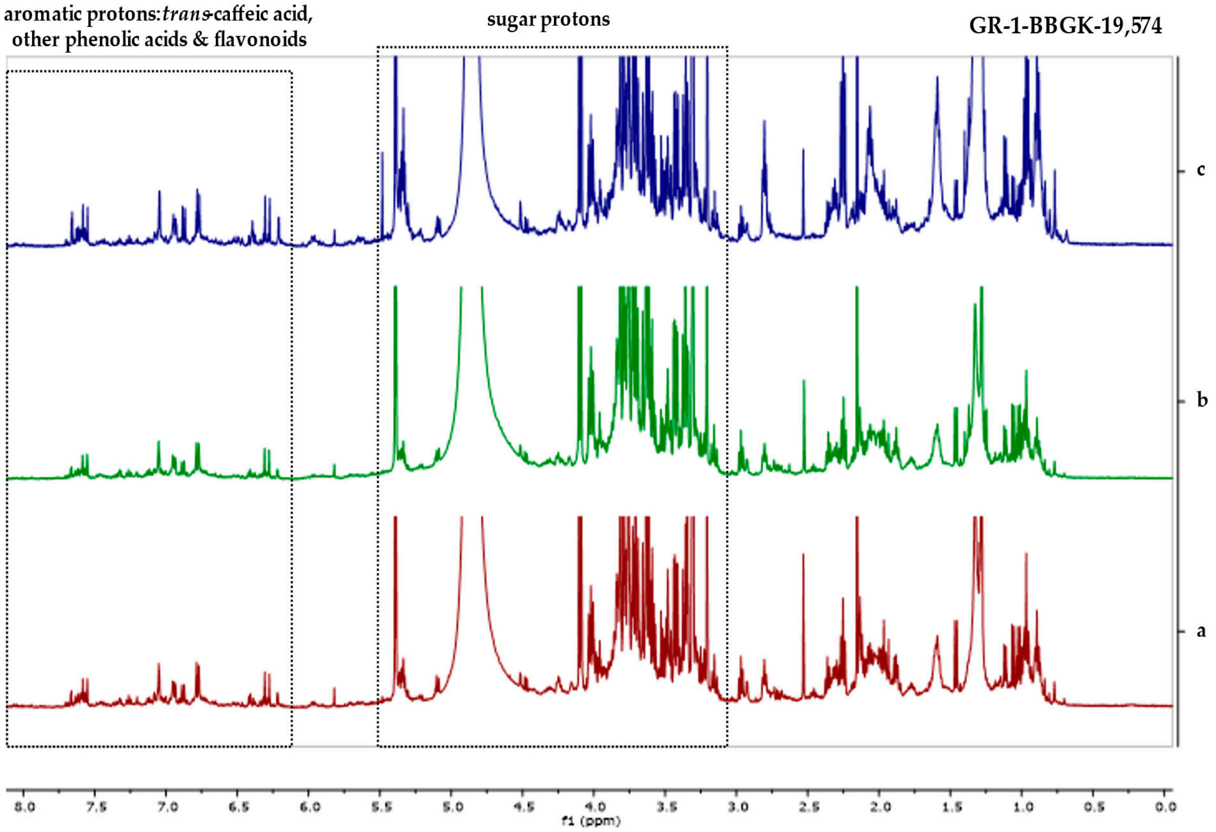

Supplementary Figure S6: ^{1}H-NMR spectra of hydromethanolic extracts of *Sambucus nigra* leaves from genotype GR-1-BBGK-19,574 under (a) no fertilization (control), (b) conventional and (c) organic fertilization treatment (CD3OD, 500 MHz). Chemical shifts are reported in ppm on the x-axis (0.0 – 8.0).

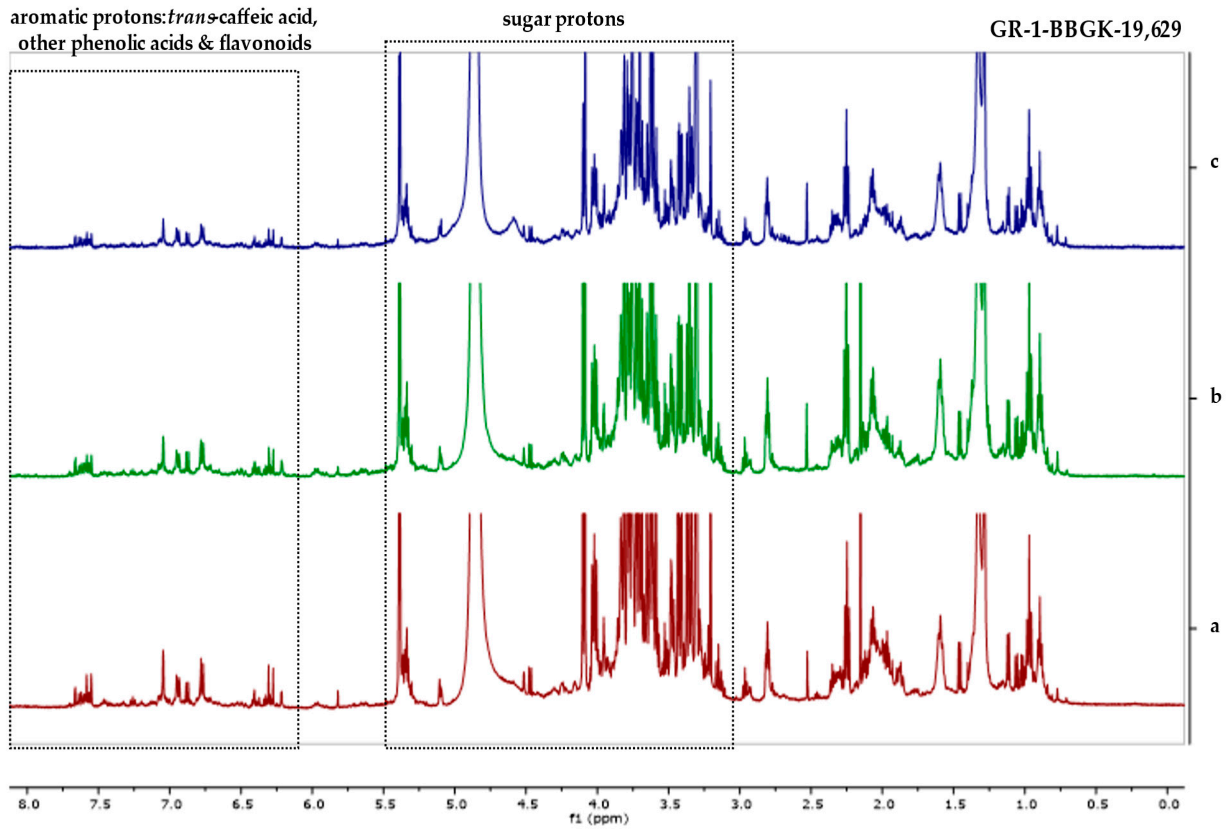

Supplementary Figure S7: ^{1}H-NMR spectra of hydromethanolic extracts of *Sambucus nigra* leaves from genotype GR-1-BBGK-19,629 under (a) no fertilization (control), (b) conventional and (c) organic fertilization treatment (CD3OD, 500 MHz). Chemical shifts are reported in ppm on the x-axis (0.0 – 8.0).

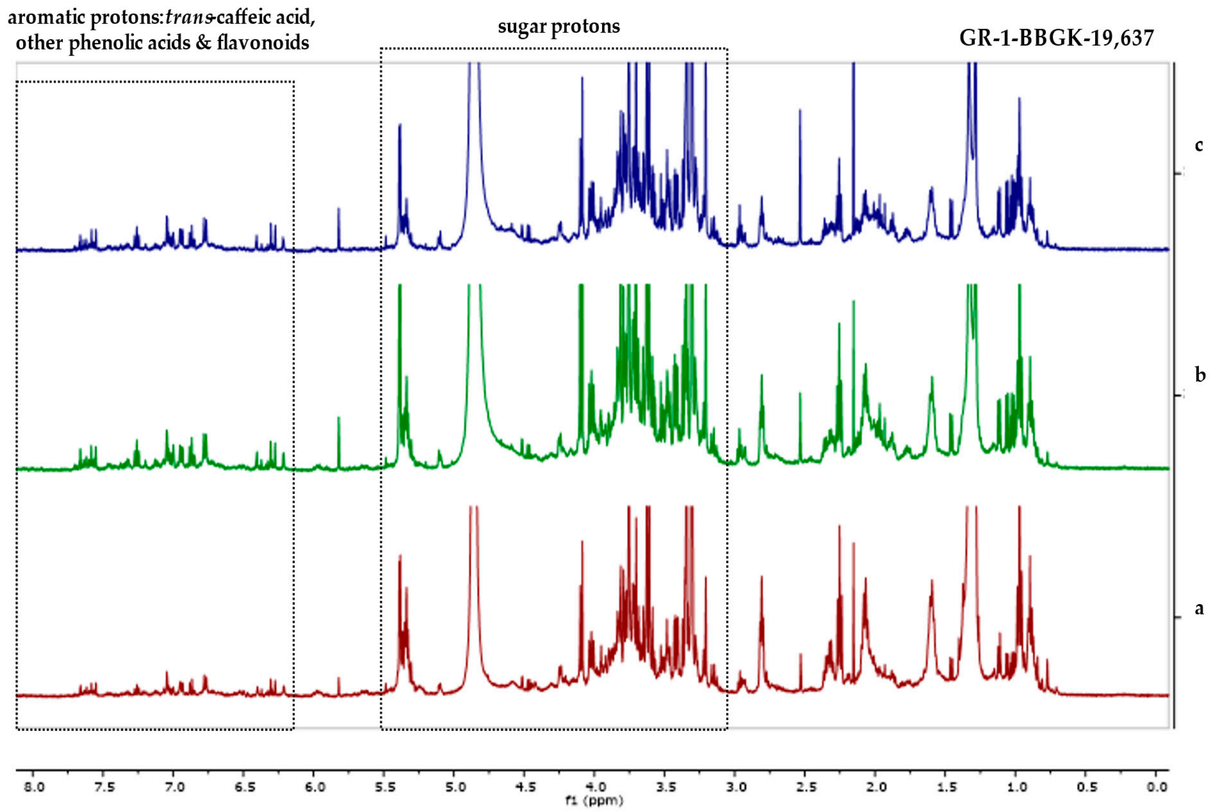

Supplementary Figure S8: ^{1}H-NMR spectra of hydromethanolic extracts of *Sambucus nigra* leaves from genotype GR-1-BBGK-19,637 under (a) no fertilization (control), (b) conventional and (c) organic fertilization treatment (CD3OD, 500 MHz). Chemical shifts are reported in ppm on the x-axis (0.0 – 8.0).

Supplementary Table S1. Wild-growing genotypes of Greek native *Sambucus nigra* sampled from wild-growing populations of northern and north-central Greece assigned with different IPEN (International Plant Exchange Network) accession numbers.

No	IPEN Accession Number	Greek Prefecture	Area	Coordinates (HGRS87/EGSA87)	Altitude (m)
1	GR-1-BBGK-19,425	Thessaly	Trikala	39.667850000000001, 21.184380000000001	1117
2	GR-1-BBGK-19,629	Epirus	Ioannina	39.787129999999998, 20.797750000000001	990
